# A Paradoxical AKT: Exploring the Promise and Challenges of PI3K/AKT/mTOR Targeted Therapies

**DOI:** 10.33696/cancerimmunol.6.089

**Published:** 2024

**Authors:** Gennie L. Parkman, Sheri L. Holmen

**Affiliations:** 1 Department of Zoology, Weber State University, Ogden, Utah 84408, USA; 2 Huntsman Cancer Institute, University of Utah Health Sciences Center, Salt Lake City, Utah 84112, USA; 3 Department of Surgery, University of Utah Health Sciences Center, Salt Lake City, Utah 84112, USA

**Keywords:** Melanoma, AKT, PI3K, mTOR, Immune system

## Introduction

Cutaneous melanoma, a malignancy originating from melanocytes in the skin, is challenging to treat due to its aggressive nature and propensity to metastasize. Despite the advancement of multifaceted treatment interventions, including targeted therapy, immunotherapy, and cellular therapy, the five-year-survival for patients with stage IV melanoma is only ~30% [1–3]. BRAF activating mutations are the most common genetic alteration in melanoma, comprising ~50% of all cutaneous melanoma cases [4,5]. Despite the prevalence of BRAF mutations in this heterogenous disease, a wealth of evidence demonstrates that this alteration is insufficient to promote malignancy without additional genetic abnormalities [6,7]. Thus, the activation of other cellular signaling pathways cooperates with activation of BRAF to cause the transition from benign melanocytic nevi to malignant melanoma. Over the past few decades, substantial progress has been made in understanding the molecular pathways underlying melanoma progression and resistance mechanisms. Among these pathways, the phosphatidylinositol 3-kinase (PI3K)/protein kinase B (AKT)/mammalian target of rapamycin (mTOR) signaling cascade has emerged as a promising target for therapeutic intervention. This commentary explores the role of the PI3K/AKT/mTOR pathway in BRAF-mutant melanoma, highlighting the mechanisms of action, preclinical and clinical efficacy of treatment options aimed at this signaling pathway, resistance mechanisms, and role of the immune system to uncover research questions that may lead to therapeutic options for this heterogenous disease.

The PI3K/AKT/mTOR pathway is a central signaling cascade involved in regulating various cellular processes, including cell growth, proliferation, survival, and metabolism [8]. Dysregulation of this pathway, often through genetic mutations or aberrant signaling, contributes to the acquisition of aggressive traits in many cancers, including melanoma, enhanced proliferation and survival, as well as migration and invasion leading to metastasis [9,10]. PI3K enzymes are lipid kinases that phosphorylate phosphatidylinositol lipids, leading to the activation of downstream effectors such as AKT and mTOR. mTOR exists in two distinct complexes, mTORC1 and mTORC2, which regulate protein synthesis, autophagy, and metabolism in response to various stimuli [11].

AKT is classically considered the central signaling node of the PI3K/AKT/mTOR pathway. Upon activation by upstream stimuli, such as growth factors binding to receptor tyrosine kinases, PI3K is recruited to the cell membrane, where it phosphorylates phosphatidylinositol 4,5-bisphosphate (PIP2) to generate phosphatidylinositol 3,4,5-trisphosphate (PIP3). PIP3 serves as a second messenger that recruits AKT to the cell membrane, where it becomes activated through phosphorylation by phosphoinositide-dependent kinase 1 (PDK1) and mTORC2 [12]. Past research debates the necessity of phosphorylation by both PDK1 and mTORC2 to confer full activation of AKT, yet recent literature, including our own, has demonstrated that phosphorylation by mTORC2 at Thr308 is both sufficient and necessary for activation of this protein, while Ser473 phosphorylation is dispensable [13,14]. Upon this critical activation, AKT phosphorylates a plethora of downstream targets involved in various cellular processes, including glycogen synthesis, protein synthesis, cell cycle progression, and cell survival. Among these downstream effectors, AKT phosphorylates and inhibits tuberous sclerosis complex 2 (TSC2), a negative regulator of mTOR complex 1 (mTORC1). Inhibition of TSC2 relieves its suppression of mTORC1, leading to activation of mTORC1. Thus, AKT interacts directly with mTOR through each of its two complexes – via phosphorylation by mTORC2, which ultimately leads to activation of mTORC1 by regulating key effectors, including ribosomal protein S6 kinase (S6K) and eukaryotic translation initiation factor 4E-binding protein 1 (4E-BP1) [8,15]. In addition to its role in cancer, dysregulation of the PI3K/AKT/mTOR pathway contributes to diabetes, neurodegenerative disorders, obesity, and cardiovascular diseases.

## PI3K/AKT/mTOR Inhibitors

**Figure 1.**
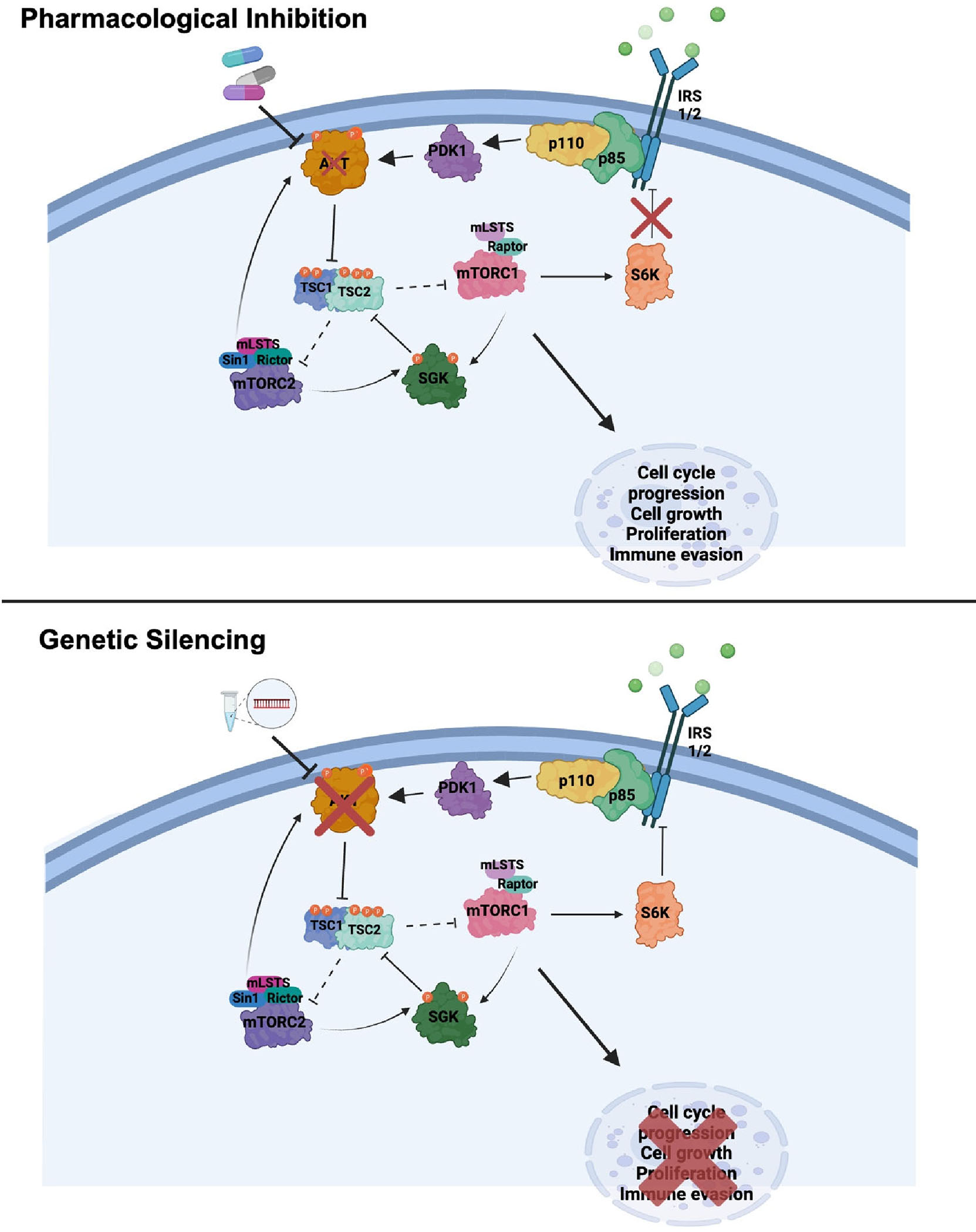
In melanoma cells, AKT activates mTORC1 promoting negative feedback. AKT pharmacological inhibitors relieve negative feedback promoting re-stimulation of the pathway through increased IRS1/2 signaling leading to cell cycle progression, cell growth, proliferation, and immune evasion dependent on downstream activation of mTORC1. siRNA-mediated genetic silencing of AKT1, 2, and 3 results in loss of AKT protein, in essence “shutting down the pathway,” resulting in cessation of signaling and thereby suppressing cell cycle progression, cell growth, proliferation, and immune evasion.

The first pharmacological inhibitor of the PI3K/AKT/mTOR pathway, rapamycin, was isolated in the 1970s from *Streptomyces hygroscopicus* bacteria and utilized as an antifungal agent [16]. It was not until the 1990s that mTOR was described as the target of rapamycin. Upon the identification of mTOR, rapamycin was studied extensively for its immunosuppressive and antiproliferative properties. This gave rise to the development of many synthetic analogs of rapamycin, termed “rapalogs” [17]. Rapamycin, and the rapalogs, primarily inhibit mTORC1, though prolonged exposure can also impact mTORC2. When mTORC1 is active, S6K phosphorylates insulin receptor substrate-1 (IRS-1), which leads to its degradation and downregulation of insulin signaling through the PI3K/AKT pathway. This negative feedback loop prevents overactivation of PI3K/AKT signaling. Inhibition of mTORC1 by rapamycin suppresses S6K activity, reducing the phosphorylation of IRS-1. This leads to stabilization and accumulation of IRS-1, enhancing its ability to activate PI3K and, subsequently, AKT (Figure 1). Therefore, relief from this negative feedback can result in activation of the PI3K/AKT pathway despite mTORC1 inhibition [18]. This paradoxical activation led to the development of newer generation mTOR inhibitors as well as PI3K inhibitors and dual PI3K/mTOR inhibitors.

PI3K inhibitors function by blocking the activity of PI3K enzymes, responsible for phosphorylation of phosphatidylinositol lipids, leading to activation of downstream effectors, notably AKT and mTOR. PI3K comprises three classes of enzymes: class I, II, and III. Furthermore, Class I PI3Ks are further subdivided into subclasses based on their structure and substrate specificity, including PI3Kα, PI3Kβ, PI3Kδ, and PI3Kγ. This dynamic class of small molecule inhibitors can target multiple or specific isoforms, of which PI3Kα is the most activated in human cancer [19,20]. Despite preclinical success of PI3K inhibitors, such as buparlisib and alpelisib, clinical trials have not revealed significant efficacy in melanoma patients, although interestingly, alpelisib has shown success in breast malignancies even with similar activation of the PI3K pathway in these cancers [21,22]. There may be potential for PI3K-isoform-specific therapy in melanoma, however. A phase 1A (NCT03544905) study evaluating CYH33, a highly selective PI3Kα inhibitor, in advanced solid tumors revealed a well-tolerated treatment with an objective response rate of 14.3%, prompting further investigation [23]. Recently, pre-clinical data demonstrated the use of a p110β inhibitor in combination with induction of RhoA, a Ras homolog family member A, significantly slowed melanoma growth, suggesting p110β inhibition may be advantageous in a combinatorial drug regimen [24]. Collectively, these results highlight the complex nature of pathway activation in different contexts.

Despite multiple streams of evidence demonstrating that AKT inhibitors suppress downstream phosphorylation through the mTOR signaling cascade, AKT inhibitors have classically had little to no effect in the treatment of melanoma especially as standalone therapies [25]. Although numerous clinical trials have tested AKT inhibitors as monotherapy in melanoma patients, there has generally been insufficient efficacy to warrant their use clinically [26]. The NCI MATCH EAY131-Y trial, however, evaluated the use of AKT inhibitor capivasertib in tumors with an AKT1^E17K^ alteration, leading to constitutive hyperactivation of AKT1, and found that 28.6% of patients had an objective response rate, and one patient with endometrial adenocarcinoma achieved a complete response, prompting optimism for use of AKT inhibition in multiple cancers with this alteration [27]. Combination therapy has shown some success, albeit limited, in other cancers as well, with the most promising trial being a phase II clinical trial evaluating ipatasertib in combination with pembrolizumab, a PD-1 inhibitor, showing promising antitumor activity and a tolerable safety profile in patients with squamous cell cancer [28]. Another study investigating capivasertib in combination with paclitaxel, a chemotherapeutic agent, demonstrated improved progression-free survival in patients with metastatic breast cancer compared with paclitaxel alone [29]. In 2023, the FDA approved capivasertib in combination with fulvestrant for adults with hormone receptor-positive, HER2-negative advanced or metastatic breast cancer that also harbor genetic alterations in PIK3CA, AKT1, or PTEN and whose cancer progressed after at least one hormone therapy or recurred within a year of finishing adjuvant therapy [30]. Overall, our understanding of the lack of efficacy of AKT inhibitors, as monotherapy or in combination therapy, in melanoma is quite limited.

AKT inhibitors aim to exert their antitumor effects through multiple mechanisms. Firstly, allosteric small molecule inhibitors bind to specific sites on AKT distinct from the ATP-binding sites, leading to conformational changes in the AKT protein and thereby inhibiting its enzymatic activity. In addition, AKT ATP-competitive inhibitors prevent the phosphorylation and activation of AKT, leading to the inhibition of downstream signaling involved in cell survival and proliferation [31]. Notably, ipatasertib, a pan-AKT ATP-competitive inhibitor with some success as a combination therapy discussed above, leads to hyperphosphorylation of AKT but simultaneously locks it in an inactive conformation by preventing the accessibility of phosphatases [32].

There is significant crosstalk between the PI3K/AKT/mTOR and other signaling pathways, particularly the MAPK pathway. Thus, inhibition of one pathway often induces resistance to therapy due to upregulation of the other [33,34]. PI3K/AKT/mTOR signaling has been shown to result in therapeutic resistance to MAPK inhibition (MAPKi) in BRAF^V600E^ mutant metastatic melanoma. Activation of focal adhesion kinase (FAK), a vital regulator of the PI3K/AKT/mTOR pathway, promotes resistance to MAPKi therapies, and use of FAK inhibitors restores sensitivity to MAPKi, serving as a potential solution to overcome adaptive resistance [35,36].

The complexity of this signaling pathway underscores the difficulty in targeting any of the nodes, thus the inability to sufficiently halt tumor growth or induce regression in the disease. In addition, cancer cells often develop resistance to evade the effects of targeted therapies. This is further compounded by their bioavailability and toxicity profiles, particularly for those drugs targeting AKT. AKT is implicated in many essential cellular processes beyond those of cancer, notably glucose and insulin metabolism; consequently, inhibition can lead to significant toxicity and intolerable side effects [37].

Despite potential initial responses to PI3K/AKT/mTOR inhibitors, primary and acquired resistance remains a significant challenge in melanoma therapy. Several mechanisms contribute to resistance, including but not limited to activation of bypass signaling pathways, genomic alterations, and relief of negative feedback loops as well as compensation. Examples in melanoma include activation of receptor tyrosine kinases, such as HER2 and IGF-1R, via activation of the Forkhead Box O (FOXO) transcription factor family, which promote the transcription of RTKs and activation of pro-survival signaling cascades [38]. In breast cancer, acquired resistance to PI3K/AKT/mTOR pathway inhibitors (e.g., capivasertib) occurs through upregulation of estrogen receptor (ER) signaling prompting reactivation of mTORC1 [39]. Just as PI3K/AKT/mTOR pathway activation can lead to MAPKi-resistance, it is well established that melanoma cells can upregulate alternative signaling pathways, such as the MAPK pathway even in the context of combined PI3K/MAPK inhibition [40,41]. Additionally, acquisition of secondary mutations or copy number alterations within the PI3K/AKT/mTOR pathway components or upstream regulators can confer resistance to MAPK and PI3K inhibitors. For example, PIK3CA mutations in metastatic melanoma significantly co-occur with genomic alterations in PTEN and AKT [42]. Even potentially more common, inhibition of one node of a signaling pathway, such as mTOR, may lead to compensatory activation of upstream or downstream signaling components, allowing melanoma cells to evade inhibition.

## Genetic Silencing of AKT

A more detailed understanding of the mechanisms underlying the inefficacy of pharmacological inhibitors of the PI3K/AKT/mTOR pathway was found through genetic suppression. Small interfering RNAs (siRNAs) silence gene expression by targeting and degrading specific messenger RNA. Pharmacological inhibitors are more likely to have off-target effects due to protein homology among members of the kinase family [43]. A critical finding in exploration of this mechanism revealed a scientific paradox whereby genetic silencing of AKT resulted in profound melanoma cell death but pharmacological inhibitors had negligible effects on melanoma cell proliferation. In our recent publication, we employed various molecular and cellular techniques to investigate the effects of AKT silencing on melanoma cell viability, allowing critical inquiry into the specific function of the three AKT paralogs. siRNAs targeted to each of the AKT paralogs were transfected into numerous BRAF-mutant melanoma cell lines. siRNA-mediated knockdown of each AKT paralog (AKT1, AKT2, and AKT3) individually had little to no effect on cell proliferation, which was also observed with both allosteric and ATP-competitive pan-AKT inhibitors. However, transfection of siRNAs against all three AKT paralogs led to complete cell lethality via increased caspase 3 and 7-mediated apoptosis [14].

Although the PI3K signaling pathway is most commonly referred to as the “PI3K/AKT/mTOR” pathway, there has been debate in the melanoma field regarding the necessity of AKT as a signaling node downstream leading to the question of whether there are other PI3K lipid effectors that may play an equally important role [44]. Based on the genetic silencing findings described above, AKT is clearly indispensable for survival in melanoma cells, and this is dependent on functional kinase activity. Consequently, we further sought to investigate the mechanisms by which these two different modes of AKT suppression influenced downstream PI3K effectors.

The expression of Proline-rich Akt substrate of 40 kDa (PRAS40), which is directly phosphorylated by Akt, is commonly used as a biomarker to validate the effects of AKT inhibition [37]. However, other postulated downstream targets, such as rpS6 and 4EBP1, are not fully suppressed upon AKT inhibition despite confirmation of on-target action [14]. This led us to question whether the PI3K pathway was signaling through AKT or other crucial regulators leading to activity downstream. Interestingly, though, the answer may simply be that AKT pharmacological inhibitors are not sufficient to “shut down” the signaling cascade due to relief of negative feedback and compensatory signaling. Indeed, genetic silencing of AKT via siRNAs targeting AKT1, 2, and 3, leading to complete loss of the proteins, causes suppression of downstream effectors of mTORC1, such as 4EBP1, p70S6K, rpS6, and mTOR itself (Figure 1) [14]. Further work by others has demonstrated that PTEN, catalyzing the degradation of PIP3 into PIP2 thereby inhibiting PI3K signaling, regulates the AP-1 transcription factor, FRA1, in an AKT-dependent manner, which is dependent on mTOR translational control [45]. This collectively suggests that AKT promotes cell viability in an mTOR-dependent manner and is a central node of this pathway in melanoma.

## Compensatory Feedback Mechanisms

Despite the reliance of the PI3K pathway on AKT, other downstream proteins have been found to be influential. For instance, serum-and-glucocorticoid kinase (SGK) may act as a compensatory signaling node in melanoma when AKT is genetically silenced [46]. SGK, which also consists of three paralogs and is highly homologous to AKT, consists of an analogous catalytic domain, and relies on similar upstream activation by PI3K and mTOR [47]. RNAi-mediated silencing of AKT1, 2, and 3 can be rescued by overexpression of activated forms of the SGK paralogs, suggesting that SGK is able to compensate for AKT loss to regulate melanoma cell signaling and proliferation when the PI3K/AKT/mTOR pathway has been fully suppressed by genetic targeting of AKT. However, unlike AKT, RNAi targeting of SGK 1, 2, and 3 did not lead to melanoma cell lethality, signifying the importance of AKT as the predominant mediator with signaling through SGK as an alternative pathway. Although pharmacological inhibitors against AKT or SGK show little effect as single agents, the combined inhibition of AKT and SGK significantly reduced cell proliferation, similar to genetic targeting of AKT, and this dual treatment was able to decrease downstream signaling through mTORC1 leading to the conclusion that the effects of both genetic targeting and combination treatment of two PI3K lipid effectors is dependent on mTOR [14].

Regardless of the inability of first generation mTOR inhibitors to effectively suppress melanoma cell proliferation in the clinic, newer generation mTOR inhibitors have been developed [48]. Second generation mTOR inhibitors (e.g., MLN0128 and HY-13328) target the kinase activity whereas third generation mTOR inhibitors (e.g., RapaLink) are bivalent mTOR inhibitors, which combine rapamycin with a second-generation mTOR kinase inhibitor. These were developed to target both mTOR complexes, leading to extinguished signaling [49]. Results with RapaLink have shown promise in preclinical studies. Dual PI3K/mTOR inhibitors have been developed to circumvent relief of negative feedback signaling but most of these compounds have failed due to toxicity [33]. Paxalisib, a dual PI3K/mTOR inhibitor, has shown promise pre-clinically and even in the context of MAPK inhibitor drug-resistant disease [14,50].

## Future Directions and Potential Solutions to Current Challenges

The melanoma field has witnessed significant efficacy of immunotherapy, including anti-CTLA-4 [51], anti-PD-1, and anti-PD-L1 immune checkpoint inhibitors, which have significantly improved overall survival in patients with advanced disease. These agents prevent the interaction between checkpoint proteins on tumor cells and T cells allowing T cells to target tumor cells for death. Most recently, the FDA approved tumor-infiltrating lymphocyte (TIL) therapy, a type of patient-specific endogenous cellular immunotherapy, for patients with advanced melanoma [52]. Among the various signaling pathways that are implicated in cancer immunology, the PI3K/AKT/mTOR pathway is a key regulator of immune cell function and tumor immune evasion.

Activation of AKT in tumor cells promotes immunosuppressive features within the tumor microenvironment, such as upregulation of immune checkpoint molecules (i.e. PD-L1) and secretion of immunosuppressive cytokines, including IL-10 and TGF-β, leading to impaired antitumor immune responses and immune evasion [53,54]. Moreover, AKT activation in immune cells, for instance T cells and dendritic cells, influences their differentiation, activation, and effector functions, thereby modulating antitumor immune responses. mTOR, particularly the mTORC1 complex, regulates the activation and differentiation of immune cells through downstream activation of critical regulators of protein synthesis, including 4EBP1 and rpS6. Activation of mTOR signaling in tumor cells promotes metabolic reprogramming, leading to enhanced nutrient uptake and utilization, as well as increased expression of immune checkpoint molecules, creating an immunosuppressive tumor microenvironment [55]. Furthermore, mTOR activation in immune cells, particularly regulatory T cells (Tregs) and myeloid-derived suppressor cells (MDSCs), promotes their immunosuppressive functions, inhibiting antitumor immune responses and promoting tumor growth [56]. Additionally, metabolic alterations driven by activation of PI3K signaling influence immune cell function and differentiation. Tumor-associated macrophages (TAMs) and dendritic cells (DCs) exhibit metabolic reprogramming by PI3K/AKT/mTOR signaling, which impacts their antigen presentation and cytokine secretion [55]. Modulation of the PI3K/AKT/mTOR pathway in immune cells may enhance their antitumor activities and potentiate cancer immunotherapy.

A recent study found that alterations in *PIK3CA, AKT1, PIK3C3, and RPTOR,* were predictive of enhanced overall survival upon ICI treatment in multiple cancers including melanoma, and attributed this to enhanced anti-tumor immunity [57], which provides further evidence of the importance of this signaling cascade in immunomodulatory effects. As there is extensive crosstalk with the PI3K/AKT/mTOR pathway and immune signaling, it is reasonable that inhibitors of the PI3K pathway are being investigated in combination with ICI and other types of immunotherapies [58]. mTOR plays a multifunctional immunomodulatory role, particularly in the differentiation of effector regulatory T cells that suppress anti-tumor immunity [55,59]. Thus, preclinical studies have shown anti-tumor activity with the combination of mTOR inhibitors, such as everolimus or temsirolimus, along with checkpoint inhibitors [60]. In addition, other studies have explored combining mTOR inhibitors with cancer vaccines in melanoma. This combination aims to enhance the immune response against tumor antigens presented by the vaccine while inhibiting mTOR-mediated immunosuppression. Although there are currently no FDA-approved combination immunotherapies with PI3K/AKT/mTOR inhibitors, clinical trials are ongoing to evaluate the safety and efficacy of multiple combination therapies in melanoma [49]. Quite possibly the most exciting results have emerged from a patient case report evaluating the effects of temsirolimus in conjunction with dual nivolumab/ipilimumab therapy to treat poorly differentiated thyroid cancer. The patient’s tumor consisted of mutations in *PTEN*, thus warranting suppression of the aberrantly activated PI3K/AKT/mTOR signaling pathway. In combination with the dual immunotherapy combination, the patient experienced significant clinical improvement and disease control for six months despite previously failing multiple lines of therapy [61]. This study, although limited, represents an exciting avenue for a promising therapeutic strategy in cancers with genetic alterations in the PI3K/AKT/mTOR signaling pathway.

## Open Questions and Perspectives

The PI3K/AKT/mTOR pathway represents a key signaling cascade in melanoma, offering potential avenues for inhibiting tumor growth and overcoming resistance. Besides the importance of this multitudinous pathway in melanoma as delineated by key experimental findings, there is much more that remains to be discovered, including other PI3K lipid effectors that may also compensate in the context of pathway inhibition, additional negative feedback signaling, tissue-specific roles of each PI3K isoform and downstream effectors, epigenetic modulation of the pathway, as well as interactions between the PI3K/AKT/mTOR pathway and the tumor microenvironment, including neighboring immune and stromal cells, as well as the extracellular matrix.

PI3K/AKT/mTOR inhibitors, albeit showing pre-clinical efficacy, can lead to significant adverse toxic effects leading to systemic ineffectiveness [40]. As this pathway modulates cellular metabolic functions in normal cells, in addition to tumor cells, inhibition can commonly lead to metabolic abnormalities, gastrointestinal upset, and skin toxicities, likely limiting the use of these therapeutic agents [62]. Increased potency of PI3K/AKT/mTOR inhibitors may lead to better target modulation at more tolerable drug concentrations, or through alternative delivery approaches, including nanoparticles, polymer-based delivery systems, and liposomal formulations.

Efforts to overcome resistance and enhance the efficacy of PI3K/AKT/mTOR inhibitors in melanoma therapy are ongoing. Combination strategies involving dual pathway inhibition, immunotherapy, or targeted agents against resistance mechanisms are being explored in preclinical and clinical studies. Moreover, the identification of predictive biomarkers of response and resistance, such as genetic alterations or gene expression signatures, holds promise for patient stratification and personalized treatment approaches. While preclinical and early clinical data support the efficacy of PI3K/AKT/mTOR inhibitors in melanoma patients, challenges such as limited suppression of the pathway and toxicity [34] concerns continue to warrant further investigation. Continued research efforts aimed at elucidating the complex interplay within the PI3K/AKT/mTOR pathway and developing rational combination strategies hold the key to improving outcomes for cancer patients in the future.

## References

[1] Larkin J, Chiarion-Sileni V, Gonzalez R, Grob JJ, Rutkowski P, Lao CD, Five-Year Survival with Combined Nivolumab and Ipilimumab in Advanced Melanoma. N Engl J Med. 2019;381(16):1535–46. 31562797 10.1056/NEJMoa1910836

[2] Surveillance Research Program NCI. Surveillance, Epidemiology, and End Results (SEER) Explorer: An interactive website for SEER cancer statistics 2023. Available from: https://seer.cancer.gov/statistics-network/explorer/.

[3] Robert C, Grob JJ, Stroyakovskiy D, Karaszewska B, Hauschild A, Levchenko E, Five-Year Outcomes with Dabrafenib plus Trametinib in Metastatic Melanoma. N Engl J Med. 2019;381(7):626–36. 31166680 10.1056/NEJMoa1904059

[4] Ascierto PA, Kirkwood JM, Grob JJ, Simeone E, Grimaldi AM, Maio M, The role of BRAF V600 mutation in melanoma. J Transl Med. 2012;10:85. 22554099 10.1186/1479-5876-10-85 PMC3391993

[5] Castellani G, Buccarelli M, Arasi MB, Rossi S, Pisanu ME, Bellenghi M, BRAF Mutations in Melanoma: Biological Aspects, Therapeutic Implications, and Circulating Biomarkers. Cancers (Basel). 2023;15(16):4026. 37627054 10.3390/cancers15164026 PMC10452867

[6] Pollock PM, Harper UL, Hansen KS, Yudt LM, Stark M, Robbins CM, High frequency of BRAF mutations in nevi. Nat Genet. 2003;33(1):19–20. 12447372 10.1038/ng1054

[7] Sasaki Y, Niu C, Makino R, Kudo C, Sun C, Watanabe H, BRAF point mutations in primary melanoma show different prevalences by subtype. J Invest Dermatol. 2004;123(1):177–83. 15191558 10.1111/j.0022-202X.2004.22722.x

[8] Parkman GL, Foth M, Kircher DA, Holmen SL, McMahon M. The role of PI3’-lipid signalling in melanoma initiation, progression and maintenance. Exp Dermatol. 2022;31(1):43–56. 34717019 10.1111/exd.14489 PMC8724390

[9] Cho JH, Robinson JP, Arave RA, Burnett WJ, Kircher DA, Chen G, AKT1 Activation Promotes Development of Melanoma Metastases. Cell Rep. 2015;13(5):898–905. 26565903 10.1016/j.celrep.2015.09.057 PMC4646731

[10] Kircher DA, Trombetti KA, Silvis MR, Parkman GL, Fischer GM, Angel SN, AKT1(E17K) Activates Focal Adhesion Kinase and Promotes Melanoma Brain Metastasis. Mol Cancer Res. 2019;17(9):1787–800. 31138602 10.1158/1541-7786.MCR-18-1372 PMC6726552

[11] Liu P, Cheng H, Roberts TM, Zhao JJ. Targeting the phosphoinositide 3-kinase pathway in cancer. Nat Rev Drug Discov. 2009;8(8):627–44. 19644473 10.1038/nrd2926 PMC3142564

[12] Manning BD, Tee AR, Logsdon MN, Blenis J, Cantley LC. Identification of the tuberous sclerosis complex-2 tumor suppressor gene product tuberin as a target of the phosphoinositide 3-kinase/akt pathway. Mol Cell. 2002;10(1):151–62. 12150915 10.1016/s1097-2765(02)00568-3

[13] Vivanco I, Sawyers CL. The phosphatidylinositol 3-Kinase AKT pathway in human cancer. Nat Rev Cancer. 2002;2(7):489–501. 12094235 10.1038/nrc839

[14] Parkman GL, Turapov T, Kircher DA, Burnett WJ, Stehn CM, O’Toole K, Genetic Silencing of AKT Induces Melanoma Cell Death via mTOR Suppression. Mol Cancer Ther. 2024;23(3):301–15. 37931033 10.1158/1535-7163.MCT-23-0474 PMC10932877

[15] Kwong LN, Davies MA. Navigating the therapeutic complexity of PI3K pathway inhibition in melanoma. Clin Cancer Res. 2013;19(19):5310–9. 24089444 10.1158/1078-0432.CCR-13-0142 PMC3811159

[16] Rapamycin Seto B. and mTOR: a serendipitous discovery and implications for breast cancer. Clin Transl Med. 2012;1(1):29. 23369283 10.1186/2001-1326-1-29 PMC3561035

[17] Thomson AW, Turnquist HR, Raimondi G. Immunoregulatory functions of mTOR inhibition. Nat Rev Immunol. 2009;9(5):324–37. 19390566 10.1038/nri2546 PMC2847476

[18] O’Reilly KE, Rojo F, She QB, Solit D, Mills GB, Smith D, mTOR inhibition induces upstream receptor tyrosine kinase signaling and activates Akt. Cancer Res. 2006;66(3):1500–8. 16452206 10.1158/0008-5472.CAN-05-2925 PMC3193604

[19] Samuels Y, Diaz LA Jr., Schmidt-Kittler O, Cummins JM, Delong L, Cheong I, Mutant PIK3CA promotes cell growth and invasion of human cancer cells. Cancer Cell. 2005;7(6):561–73. 15950905 10.1016/j.ccr.2005.05.014

[20] Fruman DA, Chiu H, Hopkins BD, Bagrodia S, Cantley LC, Abraham RT. The PI3K Pathway in Human Disease. Cell. 2017;170(4):605–35. 28802037 10.1016/j.cell.2017.07.029 PMC5726441

[21] Chang DY, Ma WL, Lu YS. Role of Alpelisib in the Treatment of PIK3CA-Mutated Breast Cancer: Patient Selection and Clinical Perspectives. Ther Clin Risk Manag. 2021;17:193–207. 33707948 10.2147/TCRM.S251668 PMC7943556

[22] Rodon J, Brana I, Siu LL, De Jonge MJ, Homji N, Mills D, Phase I dose-escalation and -expansion study of buparlisib (BKM120), an oral pan-Class I PI3K inhibitor, in patients with advanced solid tumors. Invest New Drugs. 2014;32(4):670–81. 24652201 10.1007/s10637-014-0082-9

[23] Wei XL, Liu FR, Liu JH, Zhao HY, Zhang Y, Wang ZQ, First-in-human phase Ia study of the PI3Kalpha inhibitor CYH33 in patients with solid tumors. Nat Commun. 2022;13(1):7012. 36385120 10.1038/s41467-022-34782-9 PMC9669016

[24] Tzenaki N, Xenou L, Goulielmaki E, Tsapara A, Voudouri I, Antoniou A, A combined opposite targeting of p110delta PI3K and RhoA abrogates skin cancer. Commun Biol. 2024;7(1):26. 38182748 10.1038/s42003-023-05639-8 PMC10770346

[25] Marsh Durban V, Deuker MM, Bosenberg MW, Phillips W, McMahon M. Differential AKT dependency displayed by mouse models of BRAFV600E-initiated melanoma. J Clin Invest. 2013;123(12):5104–18. 24200692 10.1172/JCI69619 PMC3859393

[26] Brown JS, Banerji U. Maximising the potential of AKT inhibitors as anti-cancer treatments. Pharmacol Ther. 2017;172:101–15. 27919797 10.1016/j.pharmthera.2016.12.001 PMC6143165

[27] Kalinsky K, Hong F, McCourt CK, Sachdev JC, Mitchell EP, Zwiebel JA, Effect of Capivasertib in Patients With an AKT1 E17K-Mutated Tumor: NCI-MATCH Subprotocol EAY131-Y Nonrandomized Trial. JAMA Oncol. 2021;7(2):271–8. 33377972 10.1001/jamaoncol.2020.6741 PMC7774047

[28] Colevas JSTRHVVJRJNMKSGD. Abstract CT047: A phase 2 study of ipatasertib in combination with pembrolizumab for first line treatment of recurrent or metastatic squamous cell cancer of the head and neck. Cancer Research. 2023;83.

[29] Schmid P, Abraham J, Chan S, Wheatley D, Brunt AM, Nemsadze G, Capivasertib Plus Paclitaxel Versus Placebo Plus Paclitaxel As First-Line Therapy for Metastatic Triple-Negative Breast Cancer: The PAKT Trial. J Clin Oncol. 2020;38(5):423–33. 31841354 10.1200/JCO.19.00368

[30] Administration USFD. FDA approves capivasertib with fulvestrant for breast cancer 2023 [Available from: https://www.fda.gov/drugs/resources-information-approved-drugs/fda-approves-capivasertib-fulvestrant-breast-cancer. 10.1002/cncr.35238 38396318

[31] Lazaro G, Kostaras E, Vivanco I. Inhibitors in AKTion: ATP-competitive vs allosteric. Biochem Soc Trans. 2020;48(3):933–43. 32453400 10.1042/BST20190777 PMC7329346

[32] Saura C, Roda D, Rosello S, Oliveira M, Macarulla T, Perez-Fidalgo JA, A First-in-Human Phase I Study of the ATP-Competitive AKT Inhibitor Ipatasertib Demonstrates Robust and Safe Targeting of AKT in Patients with Solid Tumors. Cancer Discov. 2017;7(1):102–13. 27872130 10.1158/2159-8290.CD-16-0512 PMC5463454

[33] Mendoza MC, Er EE, Blenis J. The Ras-ERK and PI3K-mTOR pathways: cross-talk and compensation. Trends Biochem Sci. 2011;36(6):320–8. 21531565 10.1016/j.tibs.2011.03.006 PMC3112285

[34] Chen B, Tardell C, Higgins B, Packman K, Boylan JF, Niu H. BRAFV600E negatively regulates the AKT pathway in melanoma cell lines. PLoS One. 2012;7(8):e42598. 22880048 10.1371/journal.pone.0042598 PMC3411810

[35] Di Leo L, Pagliuca C, Kishk A, Rizza S, Tsiavou C, Pecorari C, AMBRA1 levels predict resistance to MAPK inhibitors in melanoma. Proc Natl Acad Sci U S A. 2024;121(25):e2400566121. 38870061 10.1073/pnas.2400566121 PMC11194594

[36] Hirata E, Girotti MR, Viros A, Hooper S, Spencer-Dene B, Matsuda M, Intravital imaging reveals how BRAF inhibition generates drug-tolerant microenvironments with high integrin beta1/FAK signaling. Cancer Cell. 2015;27(4):574–88. 25873177 10.1016/j.ccell.2015.03.008 PMC4402404

[37] Coleman N, Moyers JT, Harbery A, Vivanco I, Yap TA. Clinical Development of AKT Inhibitors and Associated Predictive Biomarkers to Guide Patient Treatment in Cancer Medicine. Pharmgenomics Pers Med. 2021;14:1517–35. 34858045 10.2147/PGPM.S305068 PMC8630372

[38] He Y, Sun MM, Zhang GG, Yang J, Chen KS, Xu WW, Targeting PI3K/Akt signal transduction for cancer therapy. Signal Transduct Target Ther. 2021;6(1):425. 34916492 10.1038/s41392-021-00828-5 PMC8677728

[39] Turner SMAPL-XSRCPSHSAGSWSBED-BNC. Abstract LB450: Mechanisms of acquired resistance to AKT inhibitor capivasertib in AKT1 mutant patient derived breast cancer models. Cancer Res. 2024;84.37874330

[40] McNeill RS, Canoutas DA, Stuhlmiller TJ, Dhruv HD, Irvin DM, Bash RE, Combination therapy with potent PI3K and MAPK inhibitors overcomes adaptive kinome resistance to single agents in preclinical models of glioblastoma. Neuro Oncol. 2017;19(11):1469–80. 28379424 10.1093/neuonc/nox044 PMC5737415

[41] Murali I, Kasar S, Naeem A, Tyekucheva S, Khalsa JK, Thrash EM, Activation of the MAPK pathway mediates resistance to PI3K inhibitors in chronic lymphocytic leukemia. Blood. 2021;138(1):44–56. 33684943 10.1182/blood.2020006765 PMC8493976

[42] cBioPortal. 2024.

[43] Weiss WA, Taylor SS, Shokat KM. Recognizing and exploiting differences between RNAi and small-molecule inhibitors. Nat Chem Biol. 2007;3(12):739–44. 18007642 10.1038/nchembio1207-739 PMC2924165

[44] Silva JM, Bulman C, McMahon M. BRAFV600E cooperates with PI3K signaling, independent of AKT, to regulate melanoma cell proliferation. Mol Cancer Res. 2014;12(3):447–63. 24425783 10.1158/1541-7786.MCR-13-0224-T PMC3966216

[45] Xu X, Bok I, Jasani N, Wang K, Chadourne M, Mecozzi N, PTEN Lipid Phosphatase Activity Suppresses Melanoma Formation by Opposing an AKT/mTOR/FRA1 Signaling Axis. Cancer Res. 2024;84(3):388–404. 38193852 10.1158/0008-5472.CAN-23-1730 PMC10842853

[46] Orlacchio A, Ranieri M, Brave M, Arciuch VA, Forde T, De Martino D, SGK1 Is a Critical Component of an AKT-Independent Pathway Essential for PI3K-Mediated Tumor Development and Maintenance. Cancer Res. 2017;77(24):6914–26. 29055016 10.1158/0008-5472.CAN-17-2105 PMC5732884

[47] Bruhn MA, Pearson RB, Hannan RD, Sheppard KE. Second AKT: the rise of SGK in cancer signalling. Growth Factors. 2010;28(6):394–408. 20919962 10.3109/08977194.2010.518616

[48] Bouyahya A, El Allam A, Aboulaghras S, Bakrim S, El Menyiy N, Alshahrani MM, Targeting mTOR as a Cancer Therapy: Recent Advances in Natural Bioactive Compounds and Immunotherapy. Cancers (Basel). 2022;14(22):5520. 36428613 10.3390/cancers14225520 PMC9688668

[49] Mir SA, Dar A, Alshehri SA, Wahab S, Hamid L, Almoyad MAA, Exploring the mTOR Signalling Pathway and Its Inhibitory Scope in Cancer. Pharmaceuticals (Basel). 2023;16(7):1004. 37513916 10.3390/ph16071004 PMC10384750

[50] ClinicalTrials.gov. Study of GDC-0084 in pediatric patients with newly diagnosed diffuse intrinsic pontine glioma or diffuse midline gliomas. 2022. Available from: https://clinicaltrials.gov/ct2/show/NCT03696355.

[51] Mansh M Ipilimumab and cancer immunotherapy: a new hope for advanced stage melanoma. Yale J Biol Med. 2011;84(4):381–9. 22180676 PMC3238313

[52] Phillips C First Cancer TIL Therapy Gets FDA Approval for Advanced Melanoma 2024 [Available from: https://www.cancer.gov/news-events/cancer-currents-blog/2024/fda-amtagvi-til-therapy-melanoma.

[53] Lin NSPMY-PJLBKPCLAvdVR. Abstract 522: PI3K/Akt signaling in tumor cells promotes immune evasion by limiting infiltration, recognition and killing by T cells. Cancer Research. 2019;79.31641034

[54] Guerau-de-Arellano M, Piedra-Quintero ZL, Tsichlis PN. Akt isoforms in the immune system. Front Immunol. 2022;13:990874. 36081513 10.3389/fimmu.2022.990874 PMC9445622

[55] Mafi S, Mansoori B, Taeb S, Sadeghi H, Abbasi R, Cho WC, mTOR-Mediated Regulation of Immune Responses in Cancer and Tumor Microenvironment. Front Immunol. 2021;12:774103. 35250965 10.3389/fimmu.2021.774103 PMC8894239

[56] Weichhart T, Hengstschlager M, Linke M. Regulation of innate immune cell function by mTOR. Nat Rev Immunol. 2015;15(10):599–614. 26403194 10.1038/nri3901 PMC6095456

[57] Cheng L, Wang Y, Qiu L, Chang Y, Lu H, Liu C, mTOR pathway gene mutations predict response to immune checkpoint inhibitors in multiple cancers. J Transl Med. 2022;20(1):247. 35642038 10.1186/s12967-022-03436-1 PMC9153162

[58] Sirico M, D’Angelo A, Gianni C, Casadei C, Merloni F, De Giorgi U. Current State and Future Challenges for PI3K Inhibitors in Cancer Therapy. Cancers (Basel). 2023;15(3):703. 36765661 10.3390/cancers15030703 PMC9913212

[59] Zeng H, Chi H. mTOR signaling in the differentiation and function of regulatory and effector T cells. Curr Opin Immunol. 2017;46:103–11. 28535458 10.1016/j.coi.2017.04.005 PMC5554750

[60] El Hage A, Dormond O. Combining mTOR Inhibitors and T Cell-Based Immunotherapies in Cancer Treatment. Cancers (Basel). 2021;13(6):1359. 33802831 10.3390/cancers13061359 PMC8002586

[61] Oh Y, Park JH, Djunadi TA, Shah Z, Chung LI, Chae YK. Deep response to a combination of mTOR inhibitor temsirolimus and dual immunotherapy of nivolumab/ipilimumab in poorly differentiated thyroid carcinoma with PTEN mutation: a case report and literature review. Front Endocrinol (Lausanne). 2024;15:1304188. 38356955 10.3389/fendo.2024.1304188 PMC10864638

[62] Breal C, Beuvon F, de Witasse-Thezy T, Dermine S, Franchi-Rezgui P, Deau-Fisher B, Management of Gastro-Intestinal Toxicity of the Pi3 Kinase Inhibitor: Optimizing Future Dosing Strategies. Cancers (Basel). 2023;15(8):2279. 37190206 10.3390/cancers15082279 PMC10136502

